# Rare variant collapsing in conjunction with mean log *p*-value and gradient boosting approaches applied to Genetic Analysis Workshop 17 data

**DOI:** 10.1186/1753-6561-5-S9-S94

**Published:** 2011-11-29

**Authors:** Yauheniya Cherkas, Nandini Raghavan, Stephan Francke, Frank DeFalco, Marsha A Wilcox

**Affiliations:** 1 Epidemiology, Johnson & Johnson, 1125 Trenton-Harbourton Road, Titusville, NJ 08560, USA; 2 Non-Clinical Biostatistics, Johnson & Johnson, OMP Building, 1000 Route 202-S, Raritan, NJ 08869, USA; 3 Pharmacogenomics, Johnson & Johnson PRD, PO Box 300, 1000 Route 202, Raritan, NJ 08869, USA; 4 Informatics, Johnson & Johnson, 920 Route 202, Raritan, NJ 08869, USA

## Abstract

In addition to methods that can identify common variants associated with susceptibility to common diseases, there has been increasing interest in approaches that can identify rare genetic variants. We use the simulated data provided to the participants of Genetic Analysis Workshop 17 (GAW17) to identify both rare and common single-nucleotide polymorphisms and pathways associated with disease status. We apply a rare variant collapsing approach and the usual association tests for common variants to identify candidates for further analysis using pathway-based and tree-based ensemble approaches. We use the mean log p-value approach to identify a top set of pathways and compare it to those used in simulation of GAW17 dataset. We conclude that the mean log p-value approach is able to identify those pathways in the top list and also related pathways. We also use the stochastic gradient boosting approach for the selected subset of single-nucleotide polymorphisms. When compared the result of this tree-based method with the list of single-nucleotide polymorphisms used in dataset simulation, in addition to correct SNPs we observe number of false positives.

## Background

Many genome-wide association studies (GWAS) have been conducted in the search for specific genetic variants associated with common diseases. In testing for association with common polymorphisms, those variants that were identified were able to explain a modest proportion of disease heritability. This led to the hypothesis that multiple rare variants may play a role in complex disease etiology [1][2][3]. The multiple rare variants or common disease/rare variant hypothesis states that multiple rare variants with moderate to high penetrances underlie the susceptibility to a common disease. It is likely that both common and rare genetic variants contribute to disease risk.

Approaches targeted at uncovering associations between common polymorphisms and disease are generally underpowered for detecting the influence of rare variants. To identify disease-associated rare variants, investigators have proposed several methods based on the collapsing of low-frequency single-nucleotide polymorphisms (SNPs) [4-7].

In this analysis we use the methods proposed by Li and Leal [4] and Morris and Zeggini [8] to identify rare variants, and we use association analysis to identify common variants that confer liability to disease. The rationale behind this collapsing approach is that although the probability that an individual carries more than one rare allele may be low, in aggregate rare alleles may be common enough to account for variation in common traits. The goal is then to test for an association of an accumulation of rare minor alleles with the disease trait, by combining information across multiple variant sites.

We begin our analyses with the collapsing methods and extend the analyses in two ways. First, we use the mean log *p*-value (MLP) [9], which is a method that takes into account information about SNP function and ontologic pathway. The MLP can be thought of as a way to group together SNPs by their functional implication. It was originally developed for the analysis of gene expression data for a better understanding of the underlying mechanisms. Thus, by further analyzing the results of the rare variant collapsing approach using MLP analysis, we exploit both the spatial and the functional associations of SNPs implicated in a disease. Second, we use an empirical approach, stochastic gradient boosting (SGB), to discern groups of SNPs conferring liability to disease. SGB is an ensemble tree-based method [10] that is useful for empirically detecting sets of genes associated with a disease.

## Methods

### Data

Our analyses focus on the case-control data provided to the participants of Genetic Analysis Workshop 17 [11]. We selected the first of 200 simulated replicates for analysis. We conducted the Hardy-Weinberg equilibrium test using PLINK [12] and excluded from further analysis all markers with deviations (*p*-value less than 0.0001 in control subjects). We conducted population stratification analysis by first excluding correlated markers and then using the multidimensional scaling methods in PLINK.

### Significant markers

We use the collapsing method proposed by Li and Leal [4] and Morris and Zeggini [8] to identify possible variants among rare SNPs. For this analysis we use the CCRaVAT (Case-Control Rare Variant Analysis Tool) software package [13]. The collapsing method is as follows: first, we divide the markers into groups on the basis of predefined criteria (either genes or sliding windows of defined sequence length); next, we collapse marker data based on an indicator variable that shows whether a subject carries any rare variants; and finally, using a Pearson chi-square test, we test the significance of the difference in proportions between case subjects and control subjects who carry rare variant minor alleles. When cell counts are small, we use a Fisher exact test instead.

We consider several approaches for the collapsing criterion, including gene-based collapsing and sliding windows of five different sizes (1 kb, 5 kb, 25 kb, 50 kb, and 100 kb). The resulting *p*-values are recorded for further analysis. In addition, we test the common variant SNPs using the Pearson chi-square test. Again, the resulting *p*-values are retained for further analysis.

### MLP approach

We use the MLP approach [9] to incorporate functional and pathway information about genes into our analysis. The MLP analysis was developed in the context of gene expression analysis. The idea is to first assign a statistic (e.g., a *p*-value) to each gene. The genes are mapped onto gene sets or pathways by utilizing gene annotation databases, such as the Kyoto Encyclopedia of Genes and Genomes (KEGG) [14], the Ingenuity Pathway Analysis (IPA) [15], and the Gene Ontology (GO) Biological Processes databases [16]. Permutation tests are used to determine a *p*-value for a gene set and to identify the top set of gene sets.

In our analysis, we explore both rare and common variants. To assign a *p*-value to a gene, we use the results of collapsing and association tests. Thus a gene can have multiple *p*-values associated with it, especially in the rare variant analysis, because the windows overlap. We examine several ways to assign the *p*-value to a gene in order to explore the utility of each, three for rare variants and two for common variants. For rare variants, we use (1) the *p*-value from the association test based on gene-wise collapsing or (2) the minimum *p*-value among association tests based on the collapsing within 5-kb sliding windows located in a gene. This window size is based on the preliminary explorations of varying window sizes ranging from 1 kb to 100 kb. In addition, we use (3) the mean log *p*-value among association tests based on the collapsing within 5-kb sliding windows located in a gene. For common variants, we use either (1) the minimum *p*-value among SNPs in a gene or (2) the mean log *p*-value among SNPs in a gene.

We create gene sets, consisting of groups of genes, on the basis of one of the databases (KEGG, IPA, or GO). The gene set statistic is subsequently calculated as the MLP of the gene statistics for each gene set. The permutation procedure described by Raghavan et al. [9] is used to obtain the gene set *p*-value. The gene sets are rank-ordered by *p*-value. We examine the top 20 sets and present the top 6 sets in this report.

### Stochastic gradient boosting

SGB [10] is an ensemble tree-based method that uses an independently drawn random sample of individuals and SNPs to construct a small tree, typically containing 2 to 12 terminal nodes. The tree is grown as a result of recursively partitioning a node and contributes a small portion to the overall model. Each consecutive tree is built for the prediction residuals (from all preceding trees) of an independently drawn random sample. The final SGB model and its prediction perform by combining weighted individual tree contributions, with weight being a shrinkage parameter appropriately selected to reduce overfitting.

The SGB method produces a variable importance measure that can be used to identify top predictors. For tree methods variable importance scores show the relative contribution of each of the variables to predicting the outcome. For ensemble methods, such as SGB, the variable importance scores are averaged across all trees.

We apply the SGB method, using TreeNet, developed by Salford Systems [17], to the data consisting of the first replicate of the affected phenotype, multidimensional scaling components, environmental predictors (Age, Smoke, and Sex), and SNPs. We start with a set of the top common and rare SNPs passed from the collapsing approach and association tests. We then use the set of all SNPs provided in the GAW17 dataset.

## Results

**Figure 1:**
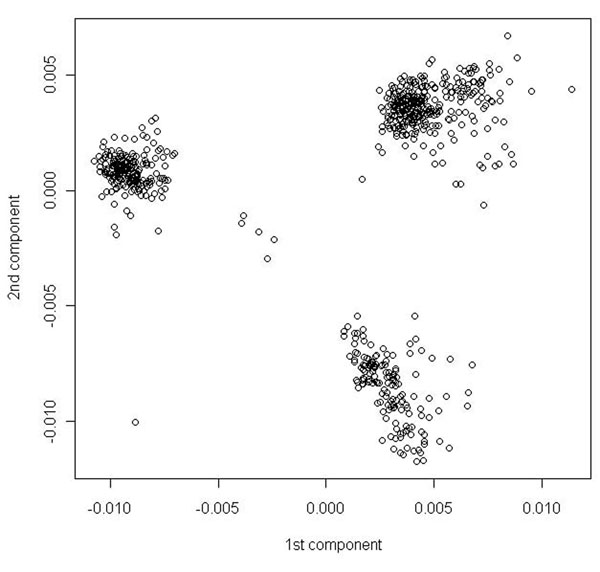
Plot of the first two components of multidimensional scaling

The initial data analysis of minor allele frequencies (MAFs) in the case-control data showed 3,224 rare variants (MAF between 1% and 5%) and 18,131 very rare variants (MAF less than 1%). There were 209 (30%) affected case subjects and 488 (70%) unaffected control subjects. Twenty-nine percent of males and thirty-one percent of females were affected. Smoking differed with case status. Smoking was prevalent in 35.9% of the affected subjects, in contrast to 21.7% of the unaffected subjects. After filtering, the data set included 22,615 SNPs. The population stratification results (the first two components) are shown in Figure 1. Three clusters were identified, corresponding to three populations. The resulting dimensions were carried forward for stratification.

**Figure 2:**
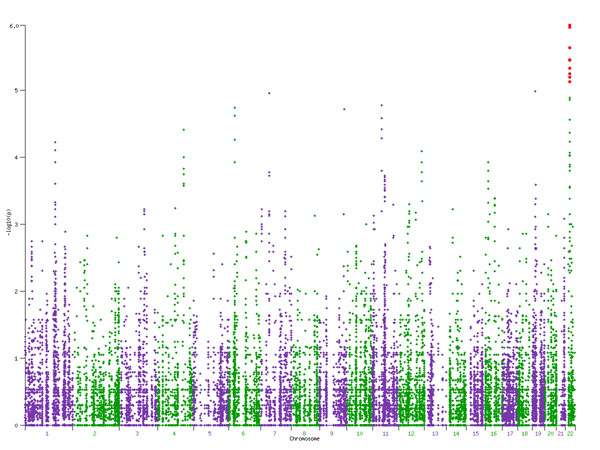
Manhattan plot of collapsing approach *p*-values for 5-kb sliding window

We performed collapsing for various window sizes (1 kb, 5 kb, 25 kb, 50 kb, and 100 kb) as well as gene-wise collapsing. The Manhattan plot of *p*-values produced by the collapsing approach for 5-kb sliding windows is shown in Figure 2. The results from the 5-kb analysis were carried into further analyses.

We used the MLP approach to identify the top gene sets based on the KEGG, IPA, and GO databases. We examined the results from the MLP analysis using IPA pathways in greater detail, because this was the database used to simulate the GAW17 data. The top 20 gene sets were examined. Results for the top six sets are summarized in Table 1. The three gene statistics for rare variants and the two gene statistics for common variants described in the “MLP Approach” subsection of the Methods section are presented. The top gene sets based on the minimum of window *p*-values shows the Notch, Hypoxia, Nitric Oxide, and vascular endothelial growth factor (VEGF) signaling pathways. Both the VEGF and the Notch signaling pathways control initiation and differentiation in angiogenesis, a process leading to blood vessel formation or remodeling. The VEGF pathway is also among the top 15 pathways identified using the KEGG database and the same statistic (results not shown).

**Table 1:** Major IPA pathways identified by the MLP approach using five statistics for rare and common variants and their corresponding *p*-values

Statistic	Top 6 pathways selected	*p*-value
Rare variants		

Gene-wise collapsing	1. Androgen and estrogen metabolism	0.0006
	2. Sphingolipid metabolism	0.0006
	3. Phenylalanine metabolism	0.0015
	4. Death receptor signaling	0.002
	5. Stilbene, coumarine, and lignin biosynthesis	0.0036
	6. TWEAK signaling	0.0043

Minimum *p*-value of 5-kb sliding window collapsing within a gene	1. Notch signaling	0.0248
	2. Hypoxia signaling in the cardiovascular system	0.0403
	3. Nitric oxide signaling in the cardiovascular system	0.0409
	4. VEGF signaling	0.0585
	5. Glutamate receptor signaling	0.0642
	6. Glutamate metabolism	0.0741

Mean log *p*-value of 5-kb sliding window collapsing within a gene	1. Cyanoamino acid metabolism	0.0032
	2. Ubiquinone biosynthesis	0.0148
	3. Nitrogen metabolism	0.0267
	4. Alanine and aspartate metabolism	0.0392
	5. GABA receptor signaling	0.0423
	6. FXR/RXR activation	0.0438

Common variants		

Minimum *p*-value among SNPs	1. Apoptosis signaling	0.0083
	2. Pyrimidine metabolism	0.0232
	3. CNTF signaling	0.0429
	4. FLT3 signaling in hematopoietic progenitor cells	0.0601
	5. Role of NANOG in mammalian embryonic stem cell pluripotency	0.0847
	6. EGF signaling	0.0908

Mean log p-value for SNPs in a gene	1. Pyrimidine metabolism	0.0021
	2. CNTF signaling	0.0127
	3. Melanocyte development and pigmentation signaling	0.0221
	4. JAK/Stat signaling	0.0327
	5. IL-15 signaling	0.0356
	6. FLT3 signaling in hematopoietic progenitor cells	0.045

We applied the SGB approach to the data containing preselected SNPs, population stratification results, and environmental variables. We used TreeNet to perform the SGB. We built 5,000 trees (iterations) with a maximum of 8 nodes. We chose a shrinkage parameter of 0.01 as appropriate for a data set of this dimension [18,19]. The top set of SNPs was selected using a variable importance measure of 7.00 as a cutoff threshold. The corresponding genes were also recorded. The results of the SGB approach are shown in Table 2. The table contains the top SNPs selected and their corresponding genes. The results that match the simulated model are shown in bold. The SGB analyses using the complete set of SNPs did not show an improvement over prior runs (results not shown).

**Table 2:** Top SNPs and corresponding genes identified using the SGB approach

	Top SNPs identified (from highest to lowest variable importance)							
SNPs	**C13S523**	C9S3621	C6S6142	C5S237	C9S4860	C22S1374	C2S5630	C11S60
	C13S905	**C1S6542**	C7S2893	C19S282	C6S1097	C10S2632	C2S955	C14S784
	C1S5779	C7S2446	C9S1469	C12S4668	C2S1087	C2S2148	C1S10506	C6S2129
	C15S3343	C12S4188	C14S1863	C2S4601	C6S2469	C10S2533	C19S609	C10S5515
	C1S5530	C17S3017	C9S1225	**C13S522**	C12S3028	C5S3461	C19S1762	C1S9584
	C19S1849	C9S4013	C22S1405	C12S622	C12S7056	C2S7558	C16S3421	C12S552
	C2S4407	C1S996	C22S1351	C20S2310	C22S1158	C15S4060	C17S1262	C3S1305
	C7S158	C10S387	C17S2377	C7S1877	C1S9718	C10S4422	C4S2872	C7S3971
	C2S689	C8S3322	C10S6566	C14S20	C7S1076	C11S3224	C1S7413	C22S146
	C8S4238	C8S4028	C18S2322	C6S6040	C12S5220	C6S6177	C19S3382	C19S2528
	C1S9506	C4S4283	C12S3528	C11S2585	C17S2376	C12S5446	C17S4841	C1S10200
	C4S2239	C7S3613	C5S4072	C11S6503	C11S4881	C1S10800	C9S123	C2S7414
	C2S1139	C3S3962	C7S3490	C10S5783	C11S1683	C9S2613	C11S2532	C7S4111
	C18S2310	C2S4079	C6S2366	C8S627	C2S6985	C1S7941	**C11S5292**	C4S4339
	C3S3938	**C6S5380**	C22S875	C1S7092	C7S2590	C11S2871	C6S2216	C6S5677
	C7S4646	C8S850	C8S271	C4S2296	C10S386	C9S5111	C15S3138	C1S7427
	C17S3510	C3S96	C22S385	C1S3900	C3S4638	C21S672	C1S1388	C10S2683
	C13S1168	C7S3697	C2S4909	C11S1280	C2S2154	C12S4591	C3S1176	C22S2039
	C11S3320	C2S873	C9S3100	C2S7390	C12S5526	C11S1599	C6S4552	C1S10256
	C10S3243	C12S5510	C4S2678	C4S2970	C2S8207	C16S560	C6S7138	C17S321
	C20S1844	C12S5445	C10S2670	C1S4009	C17S2026	C9S3554	C13S1660	C14S590
	C10S6432	C9S759	C19S4625	C1S9511	C8S934	C6S4242	C18S1560	C4S97
	C15S3744	C7S397	C19S4658	C12S4534	C9S2083	C19S5271	C7S3898	C1S4838
	C9S1607	C4S3834	C11S5644	C15S2848	C10S3777	C3S3657	C14S122	C14S3426
	C16S1482	C4S3076	C9S2542	C2S6995	C21S778	C9S1835	C15S3559	C8S3416
	C5S2032	C1S10813	C10S5690	C1S3676	C6S4400	C13S163	C22S645	C12S3039
	C1S10164	C6S7164	C22S1222	C4S649	C19S277	C1S7408	C1S1542	C4S186

Genes	*TNFRSF25*	*AHSA2*	*ADH1B*	*GPR85*	*OR13C5*	*SYTL2*	*PSME2*	*PTPRS*
	*ARHGEF10L*	*RGPD3*	*C4ORF33*	*PTPRZ1*	*CDK5RAP2*	***PDGFD***	*VTI1B*	*OR10H3*
	*KIF17*	*RGPD4*	*TKTL2*	*PAX4*	*STOM*	*EXPH5*	*BEGAIN*	*CYP4F2*
	*PDE4B*	*ACVR1C*	*ANP32C*	*SMO*	*GOLGA1*	*OR8D4*	*PAQR5*	*ZNF486*
	*PTGER3*	*LY75*	*PLEKHG4B*	*ZC3HC1*	*BRD3*	*CLEC2D*	*TSPAN3*	*NPHS1*
	*MSH4*	*PPIG*	*ZNF474*	*AKR1B1*	*CARD9*	*KLRK1*	*ADAMTS7*	*ZNF576*
	*STXBP3*	*WDR75*	*ABLIM3*	*SLC37A3*	*ECHDC3*	*OR6C1*	*ALPK3*	*LYPD5TMC4*
	*HIPK1*	*LOC729332*	*FOXI1*	*GATA4*	*ERCC6*	*OR6C65*	*SLC28A1*	*C20ORF32*
	*VTCN1*	*UGT1A10*	*PGBD1*	*TNFRSF10D*	*PGBD3*	*SRGAP1*	*AKAP13*	*PRIC285*
	***ARNT***	*COL6A3*	*BAT2*	*CDCA2*	*HKDC1*	*PLXNC1*	*ZNF213*	*PIGP*
	*ADAM15*	*MTERFD2*	*SLC44A4*	***PTK2B***	*MAT1A*	*C12ORF63*	*USP31*	*ETS2*
	*OR10J1*	*CRELD1*	*PSMB8*	*EXT1*	*CYP2C8*	*CHPT1*	*LOC100132786*	*HIRA*
	*OR10J5*	*LOC100130135*	*MDN1*	*SAMD12*	*SORCS1*	*TRAFD1*	*TRPV3*	*ARVCF*
	*UAP1*	*SETD2*	***VNN1***	*MLZE*	*CASP7*	*CAMKK2*	*KCNJ12*	*TOP3B*
	*LRRN2*	*GOLGB1*	*FUCA2*	*TG*	*DCLRE1A*	*ANAPC5*	*SLFN13*	*SUSD2*
	*NUAK2*	*TMCC1*	*UTRN*	*ANKRD15*	*TACC2*	*ZNF26*	*PIP4K2B*	*SEC14L3*
	*IKBKE*	*TRPC1*	*AGPAT4*	*PDCD1LG2*	*ATHL1*	*TNFRSF19*	*BRCA1*	*SMTN*
	*LAMB3*	*CRIPAK*	*PMS2*	*AQP3*	*GALNTL4*	***FLT1***	*FAM117A*	*LIMK2*
	*DUSP10*	*GRK4*	*TSPAN13*	*C9ORF131*	*HPS5*	*TRPC4*	*RECQL5*	*LOC100132621*
	*KIAA0133*	*AFAP1*	*NPC1L1*	*NPR2*	*NELL1*	*FREM2*	*HRH4*	
	*ARL6IP2*	*PF4V1*	*NCF1*	*POLR1E*	*OR8H1*	*PIBF1*	*B4GALT6*	
	*OXER1*	*STBD1*	*FBXO24*	*SMC5*	*OR9G4*	*RNASE6*	*MCART2*	
	*FSHR*	*FAM13A1*	*FBXL13*	*C9ORF79*	*OR4D9*	*FLJ10357*	*ZNF57*	
	*BCL11A*	*PDLIM5*	*RELN*	*ROR2*	*AHNAK*	*ACIN1*	*ZNF77*	

Boldface indicates results that match the simulated model.

The MLP approach correctly placed the VEGF signaling pathway and the two pathways related to the cardiovascular system (Hypoxia and Nitric Oxide) among the top six pathways. There are only 5 (out of 216) SNPs correctly identified using the SGB approach; their MAFs range from 0.2% to 17%. Most of the SNPs (211) placed in the top list are false positives.

## Discussion and conclusions

Traditionally, GWAS test for association of disease with common polymorphisms. Polymorphisms with population frequencies of 5% or more could be tested directly or indirectly for association with disease risk or quantitative traits, and GWAS have identified many genetic variants associated with disease traits. Replication of these results has not been consistently successful. More recently, methods have been proposed to identify multiple rare variants with small individual effect sizes that may be implicated in complex multigenic diseases. These methods are based on grouping rare variants by their physical proximity in order to combine information across them. According to Hirschhorn [20], one of the primary goals of GWAS is to discover the biologic pathways underlying polygenic diseases.

The MLP method is an effort in this direction, where both common and rare variants are considered on the basis of their functional implication in disease etiology. Our goal here was to exploit both the spatial and the functional associations of SNPs implicated in a disease to identify the underlying biologic pathways. Pathways used to simulate the affection status in the GAW17 data set were among the top four pathways identified by the MLP approach based on statistic 2 for rare variants. More specifically, the three pathways (Hypoxia, Nitric Oxide, and VEGF) were used to simulate the data. In addition, as explained in what follows, the top four pathways, including the Notch pathway, may be part of a cascade of interrelated pathways. Enriched signaling pathways in our analysis may overlap functionally and indicate processes leading to angiogenesis. Hypoxia signaling can trigger the VEGF cascade in cancer tissue angiogenesis, and the Notch processes downstream from the Hypoxia and VEGF pathways lead to a differentiation of newly formed vessels. Notch signaling can also down-regulate VEGF expression in a feedback loop. Thus the MLP approach based on statistic 2 for rare variants appears to be able to also identify related pathways and may be promising for the discovery of biological pathways implicated in disease etiology by rare variants.

One of the goals of this analysis was to compare results from a variety of functional and pathway databases and from a number of gene statistics. Our results indicate that using the IPA database, the gene statistic that identified the most relevant pathways was the minimum *p*-value derived from collapsing rare variants within 5-kb sliding windows residing in a gene. These results highlight the importance of using the most appropriate pathway database for the analysis, an aspect not explicitly discussed in the literature. Our analysis also indicates that (1) the results can be influenced by the density of coverage of rare variant SNPs in a gene; (2) gene-based collapsing may be too broad and may dilute the underlying information; and (3) using the mean of the window *p*-values may mute the signal considerably. In future work, we will evaluate alternative approaches to mapping SNP-level *p*-values to gene-level *p*-values as well as methods for combining the rare and common variants analyses.

The top pathways identified using the MLP method intersect with the pathways that contain genes from the results of the SGB approach. There are 5 correct SNPs out of 216 residing in 5 correct genes out of 188 corresponding to the top selected SNPs. The large number of false positives may be due to correlation of the SNPs identified by the SGB approach with the true SNPs used in the simulation model. Our current work includes studying methods to bridge the two approaches utilizing the functional information and the statistical correlation, respectively.

Currently, many analytic strategies rely on GWAS with one-SNP-at-a-time analyses. Although this approach has certainly yielded many promising candidates, it requires large samples to mitigate type I errors. One-SNP-at-a-time analyses generally do not take advantage of all the information present in the data, and failure of replication is commonplace. Recent attempts have been made to incorporate information from rare variants into the analysis by aggregating across SNPs that are in close proximity to each other. We have extended this further by leveraging information from SNPs that are either functionally related (MLP approach) or statistically correlated (SGB approach) with the hope of obtaining results that are credible and logically interpretable. These methods would, of course, need to be further evaluated in other data sets and other settings.

## Competing interests

The authors declare that there are no competing interests.

## Authors’ contributions

YC carried out the collapsing approach, pathway and tree-based analyses and participated in writing the manuscript. MW, NR, SF designed the analysis plan and participated in writhing the manuscript. FD prepared the data for analysis and edited the manuscript. All authors read and approved the final manuscript.
